# Combined copper and zinc deficiency is associated with reduced SARS-CoV-2 immunization response to BNT162b2 vaccination

**DOI:** 10.1016/j.heliyon.2023.e20919

**Published:** 2023-10-14

**Authors:** Thilo Samson Chillon, Kamil Demircan, Julian Hackler, Raban A. Heller, Peyman Kaghazian, Arash Moghaddam, Lutz Schomburg

**Affiliations:** a Max Rubner Center for Cardiovascular Metabolic Renal Research (CMR), Institute for Experimental Endocrinology, Charité-Universitätsmedizin Berlin, Hessische Straße 3-4, D-10115 Berlin, Germany; b Bundeswehr Hospital Berlin, Clinic of Traumatology and Orthopaedics, D-10115 Berlin, Germany; c Orthopedic and Trauma Surgery, Frohsinnstraße 12, D-63739 Aschaffenburg, Germany

**Keywords:** Trace elements, Selenium, COVID-19, Ceruloplasmin, Micronutrients

## Abstract

The essential trace elements copper, selenium and zinc are of relevance for immunity and immune response to vaccination. In this longitudinal study, adult healthcare workers (n = 126) received two doses of an mRNA vaccine (BNT162b2), and longitudinal serum samples were prepared. Vaccine-induced antibodies and their neutralizing activity were analyzed, and the trace elements copper, zinc, and selenium along with the copper transporter ceruloplasmin were measured. Subjects with combined deficiency of copper and zinc, i.e. both in the lowest tertiles at baseline, displayed particularly low antibody titers at three (Double Q1: 13 AU/mL vs. not double Q1: 29 AU/mL) and six (Double Q1: 200 AU/mL vs. not double Q1: 425 AU/mL) weeks after vaccination (p < 0.05). The results indicate the potential importance of an adequate trace element status of copper and zinc for raising a strong vaccine-induced SARS-CoV-2 antibody response, and highlights the importance of considering combined micronutrient insufficiencies, as single deficiencies may synergize.

## Highlights

• Low serum levels of copper have been associated with a reduced response to vaccination.
• Combined low copper/zinc status associated with reduced IgG titers three and six weeks post vaccination.
• It appears important to avoid trace element deficiencies during vaccination.

## Graphical abstract

**Figure:**
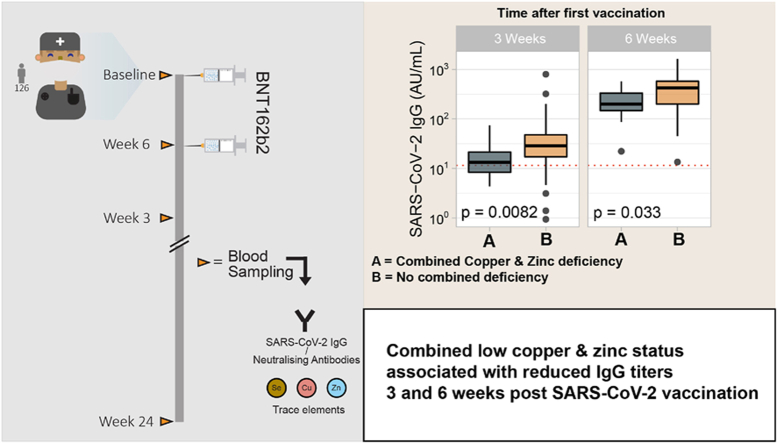

## 1 Introduction

A sufficient supply with the essential trace elements copper (Cu), selenium (Se) and zinc (Zn) is required for human development, metabolic control and maintaining health, in particular for a regular functioning of the immune system [[1], [2], [3], [4], [5]]. The trace elements Se and Zn have been identified as particularly relevant for raising a strong vaccination response, and for avoiding a severe disease course once infected [[6], [7], [8], [9], [10], [11]]. The trace element Cu serves as an important constituent of Cu-containing proteins, conferring structural stability and redox reactivity, and participates as cofactor in the enzymatic reactions catalyzed by e.g. superoxide dismutase (Cu/Zn-SOD), cytochrome *c* oxidase (COX), lysyl oxidase (LOX), peptidylglycine alpha-amidating monooxygenase (PAM), and ceruloplasmin [12,13]. Hereby, Cu and Cu-dependent enzymes contribute to many essential developmental, metabolic and adaptive biochemical pathways, with a major impact on immune system functioning and immune cell activity [[14], [15], [16], [17]].

A deficient Cu status may cause energy deficits due to insufficient activity of mitochondrial COX [18], impair regular endocrine and neuronal signaling because of low PAM expression [19], lead to elevated oxidative stress status as Cu/Zn-SOD becomes insufficiently expressed [20], and disturb regular Cu transport and iron (Fe) metabolism by insufficient Cu loading of ceruloplasmin, rendering the apoenzyme enzymatically inactive [21,22]. By these and additional mechanisms, the functionality, activity and responsiveness of immune cells depend on a regular Cu supply which is maintained by ceruloplasmin, and strong deficiency or excessive intake may impair the immune response considerably, as observed in a variety of model systems and clinical studies [14,[23], [24], [25]].

While mild to moderate Cu deficiency seems to mainly affect phagocytic and T cell activity [15,26], severe deficits were associated with leukopenia and neutropenia [14,26,27]. Dietary Cu supplementation efficiently restored some of these defects in different model systems and clinical studies [[25], [26], [27]]. In the current pandemic, the Cu status has been associated with COVID-19 severity and mortality risk [28,29], as well as with nucleic acid replication, cell entry efficiency, and virus inactivation, [[30], [31], [32], [33]]. In how far the baseline Cu status affects the immune response upon mRNA vaccination has not yet been investigated. To resolve this issue, we conducted a longitudinal observational study with adult healthcare workers who were vaccinated with an mRNA vaccine in a coordinated process during the start of the pandemic, and analyzed the interaction between Cu status and vaccination-induced humoral immune response.

## 2 Results

### 2.1 Blood sampling and baseline characteristics

**Fig. 1:**
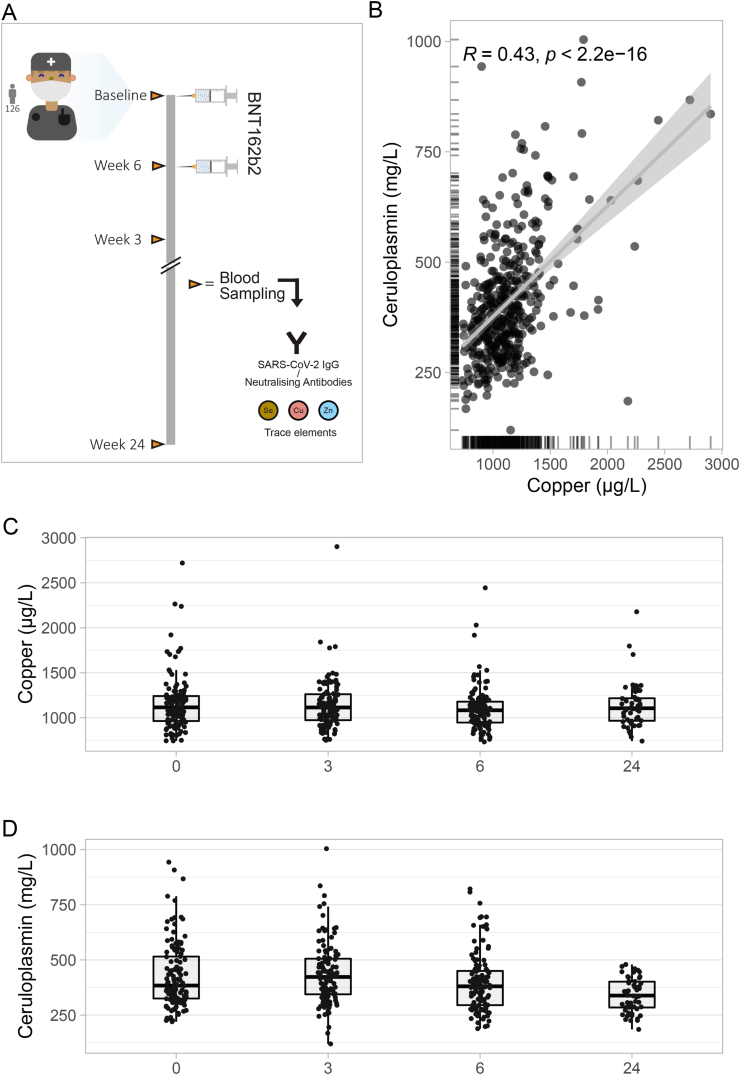
Study design, serum copper and ceruloplasmin concentrations and longitudinal dynamics. A The schematic diagram of the study design is shown. B Serum copper and ceruloplasmin concentrations show a positive linear correlation. C There are no apparent differences in serum copper concentrations during the study period. D Similarly, serum ceruloplasmin concentrations are relatively constant during the observation period. The correlation analysis was performed using Spearman's rank correlation; results are indicated as inset.

A total of 126 adult healthcare workers, of whom 110 were female and 16 were male, participated in this prospective observational study (Table 1). All participants received two doses of the BioNTech SARS-CoV-2 vaccine (BNT162b2) within a three week time interval. Four consecutive blood samples were taken, i.e., at the day of first and second vaccination, as well as 6 and 24 weeks after first vaccination (Fig. 1A).

**Table 1:** Baseline characteristics.

Characteristic	Overall, n = 126	Female, n = 110	Male, n = 16
Age (yr)	47 (37, 55)	47 (37, 55)	42 (36, 53)
SARS-CoV-2 IgG (AU/mL)	0.6 (0.0, 2.0)	0.6 (0.0, 2.0)	0.6 (0.0, 2.5)
Inhibition (%)	23 (18, 26)	23 (18, 26)	22 (14, 25)
Copper (μg/L)	1115 (963, 1241)	1161 (1013, 1252)	946 (833, 1036)
Ceruloplasmin (mg/L)	384 (325, 515)	392 (329, 534)	351 (308, 411)
Selenium (μg/L)	77 (69, 87)	76 (68, 87)	81 (73, 91)
Zinc (μg/L)	800 (738, 870)	798 (737, 869)	837 (754, 938)

Median (IQR).

At baseline all but eight of the participants showed no presence of SARS-CoV-2 Immunoglobulin G (IgG) in their serum, and concomitantly no neutralizing activity against the spike protein. The median of the IgG titer and neutralizing activity was 0.6 AU/L and 23 %, respectively. The median (IQR) concentration of serum Cu and ceruloplasmin at baseline was 1115 (963, 1241) μg/L, and 384 (325, 515) mg/L, respectively, with women displaying higher levels than men (females vs. males; Cu: 1161 (1013, 1252) vs. 946 (833, 1036) μg/L, ceruloplasmin: 392 (329, 534) vs. 351 (308, 411) mg/L). The overall median concentrations of Se and Zn were at 77 (69, 87) μg/L, and 800 (738, 870) μg/L, respectively (Table 1).

### 2.2 SARS-CoV-2 parameters and Cu status over time

The dynamics of the SARS-CoV-2 IgG concentration and neutralizing activity over the observational period of 24 weeks in this cohort was presented previously, displaying a continuous increase in the first six weeks, reaching peak values and showing subsequently declined readings at the last time point of analysis, i.e. at week 24 [13,[34], [35], [36]] (Supplementary Fig. 1). The Cu parameters total serum Cu and ceruloplasmin showed a strong linear positive correlation in the whole cohort of samples (R = 0.43, p < 0.001) (Fig. 1B). The Cu and ceruloplasmin concentrations varied slightly over the different time points of analysis (median; week 3, 6, and 24; Cu; 1114, 1083, and 1106 μg/L, versus ceruloplasmin; 423, 380, and 338 mg/L) (Fig. 1C and D).

### 2.3 Vaccination response in relation to baseline Cu status

**Fig. 2:**
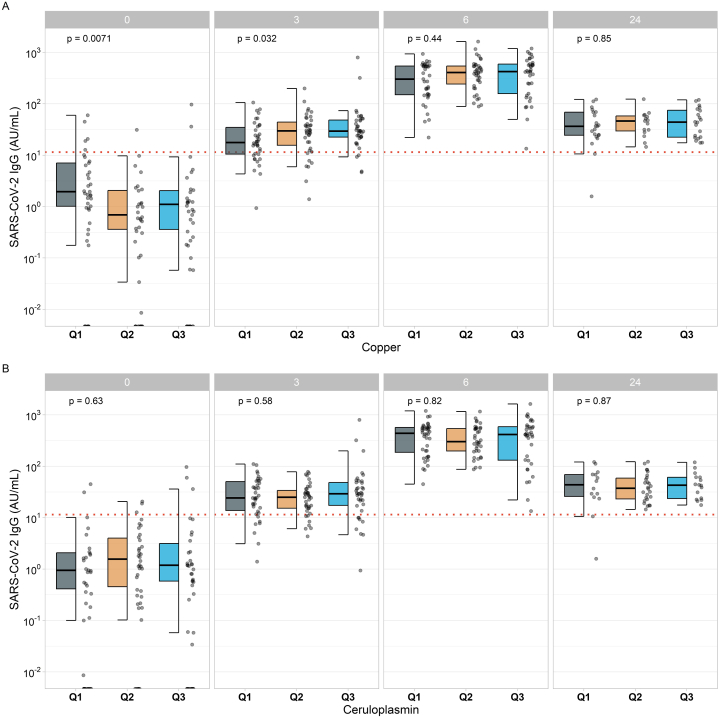
Dynamic changes in SARS-CoV-2 IgG concentrations according to baseline copper and ceruloplasmin status. A Comparison of SARS-CoV-2 IgG titers in probands at baseline and week 3, 6, and 24 post vaccination with different copper status (Q1<1024.55 μg/L; Q2 1024.55–1211 μg/L; Q3 >1211 μg/L) at baseline indicates significant differences at 3 weeks post vaccination. B The comparison of SARS-CoV-2 IgG titers in relation to baseline Ceruloplasmin status (Q1<340.70 mg/L; Q2 340.70–453.23 mg/L; Q3 >453.23 mg/L) does not reveal significant differences. Two-sided Kruskal-Wallis test was used to assess differences.

**Fig. 3:**
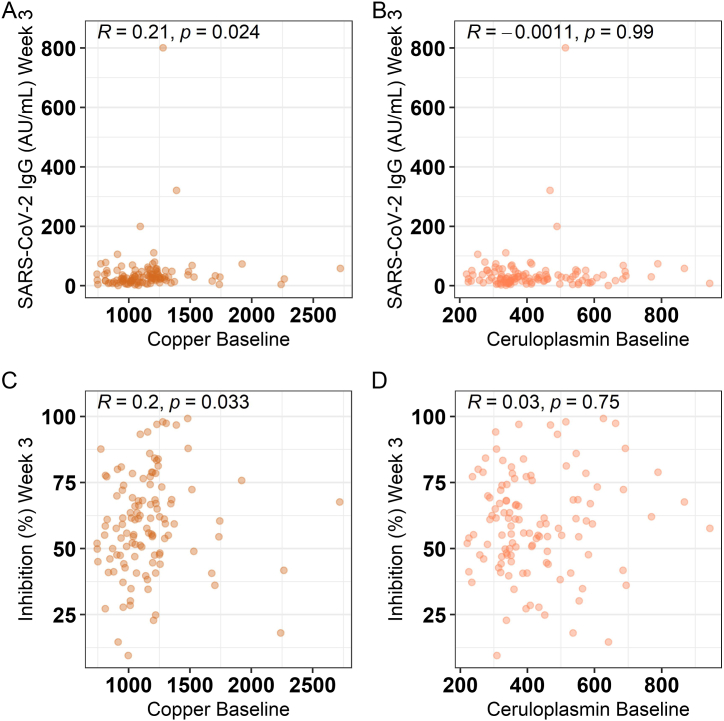
**Correlation of baseline copper and ceruloplasmin with vaccination parameters. A** Baseline copper concentration shows a linear correlation with SARS-CoV-2 IgG levels at baseline and week 3 post vaccination. **B** Baseline ceruloplasmin and SARS-CoV-2 IgG levels were not associated at week 3 post vaccination. **C** Correlation analysis of the inhibitory activity with baseline copper concentration at week 3 post vaccination indicates a moderate interaction. **D** No correlation between inhibition activity and baseline ceruloplasmin concentration at week 3 post vaccination was noted. Data were analyzed by Spearman's rank correlation.

Baseline Cu and ceruloplasmin status of the participants at first vaccination was categorized into tertiles (Cu; Q1<1024.6 μg/L; Q2: 1024.6–1211.0 μg/L; Q3>1211.0 μg/L, and ceruloplasmin; Q1<340.7 mg/L, Q2: 340.7–453.2 mg/L, Q3>453.2 mg/L). The resulting SARS-CoV-2 IgG concentrations differed significantly between the tertiles of Cu three weeks after vaccination (p < 0.05) (Fig. 2A; Supplementary Fig. 2A). A sub-analysis reveals a significant difference among the female participants only (Supplementary Fig. 3). The relatively lowest concentration of SARS-COV-2 IgG (median; 17.7 AU/mL) and neutralizing activity (median; 51.4%) was found in the samples with low Cu concentrations (Cu in Q1). In comparison, no significant differences across the tertiles of ceruloplasmin were detected (Fig. 2B; Supplementary Fig. 2B). The observed association of low Cu with low IgG concentrations and low neutralizing activity to SARS-COV-2 at the three week time point was dose-dependent, and a significant correlation was observed (p < 0.05) (Fig. 3A, C). In comparison, no correlation was detected for baseline ceruloplasmin with the IgG concentrations to SARS-COV-2 (Fig. 3B, D).

### 2.4 Vaccination response in subjects with combined trace elements deficiencies

**Fig. 4:**
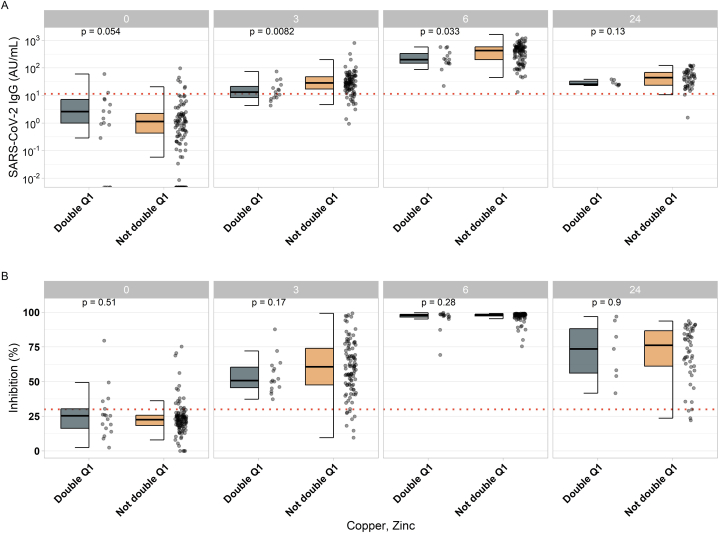
SARS-CoV-2 IgG according to double Q1 (deficiency) of copper and zinc at baseline. A Comparison of SARS-CoV-2 IgG titers in probands of different baseline copper and zinc status (Double Q1: copper <1024.55; zinc <764.3) indicates significant differences at 3 and 6 weeks post vaccination. B Comparison of neutralizing activity of different baseline copper and zinc status (Double Q1: copper <1024.55; zinc <764.3) baseline indicates no significant differences. Pairwise comparisons were conducted by applying the Wilcoxon-Rank-Sum test.

In a second analysis, participants were grouped into different categories according to combined trace element deficiencies (Table 2). Subjects who had low concentrations (Q1) of all three trace elements in parallel (Cu < 1024.6; Zn < 764.3; Se < 70.8 μg/L) were denoted as triple deficient (Triple Q1; TQ1). Participants with two trace elements in Q1 were denoted as double deficient (Double Q1, DQ1), i.e., with serum concentrations in tertile Q1 of both Cu and Zn, or Cu and Se, or Se and Zn. The comparison of these groups showed no significant associations of the triple deficiency with Cu, Se and Zn in Q1 (TQ1-Cu-Se-Zn), or of the double deficiencies in Cu and Se (DQ1-Cu-Se; Cu < 1024.6 and Se < 70.8 μg/L), or Se and Zn (DQ1-Se-Zn; Se < 70.8 and Zn < 764.3 μg/L) with the induced IgG to SARS-CoV-2 (Supplementary Figs. 4A, B, C). However, the combined double deficiency in Cu and Zn (DQ1-Cu-Zn; Cu < 1024.6 and Zn < 764.3 μg/L) was associated with significantly lower IgG concentrations to SARS-CoV-2 at weeks three and six as compared to the other subjects with no deficiency in Cu or Zn (Fig. 4A). The neutralizing activity showed no significantly differences in this comparison (Fig. 4B, Supplementary Figs. 5A and B).

**Table 2:** Group characteristics.

Group	n =	Age (yr)	Copper (μg/L)	Selenium (μg/l)	Zinc (μg/L)
**Overall**	126	47 (23–69)	1115 (743-2720)	77 (49–167)	800 (591-1208)
**Triple Q1:** (Cu, Se, Zn)	6	40 (34–49)	951 (743-1023)	55 (49–70)	665 (609–745)
**Double Q1:** (Cu, Zn)	17	44 (24–55)	917 (743-1023)	76 (49–167)	720 (592–764)
**Double Q1:** (Cu, Se)	12	40 (24–49)	943 (743-1023)	58 (49–70)	760 (609–870)
**Double Q1:** (Se, Zn)	16	42 (24–63)	1050 (743-1735)	63 (49–70)	685 (591–753)

Median (Range); Copper = Cu; Selenium = Se; Zinc = Zn.

## 3 Discussion

In this prospective observational study, we investigated the association of baseline Cu status with the humoral immune response to SARS-CoV-2 vaccination. To this end, two biomarkers of Cu status, namely total serum Cu and circulating ceruloplasmin concentrations, along with two biomarkers of vaccine-induced antibody response, namely IgG concentration and the neutralizing activity, were assessed at different time points after vaccination. The results indicate that the subjects with relatively low Cu concentrations at baseline displayed the lowest antibody response three weeks after the vaccination, whereby the effect was observed with the female participants only. However, this may be due to the relatively low number of male participants. The effect on the immune response of the group with the lowest Cu concentrations was attenuated six and 24 weeks after the first vaccination, implying that the impact may be transient in nature. A combined relative deficiency in both Cu and Zn was associated with a poor antibody response over a longer period of time, detected at 3 and 6 weeks after vaccination. Nearly half of the participants in this combined relative deficiency group tested negative for SARS-CoV-2 antibodies at the time of the second vaccination, implying a potentially greater transient vulnerability to infection as compared to the other vaccinated subjects. These participants displayed an impaired immune response, with some similarity to the response of senior (>66 yr) immunosuppressed individuals, as reported in an earlier study [37]. Other potential confounding parameters, such as baseline socio-demographic, clinical or exposure characteristics, vaccine choice or country, proved to be of little relevance for an efficient SARS-CoV-2 vaccination response [38]. In comparison to Cu, there was no parallel association between ceruloplasmin concentration and Ab response, highlighting that despite the linear correlation of both Cu biomarkers, their interaction with the immune system is apparently dissimilar, with apparently little relevance of ceruloplasmin-bound Cu within the concentration range present in this study.

The range of trace element concentrations determined in the participants of this study cohort is consistent with other recently published observational analyses with healthy European adults [[39], [40], [41]]. A significant sex-specific difference in serum Cu and ceruloplasmin was observed, in agreement with prior studies comparing healthy adult men and women [[41], [42], [43]], which is likely due to the effects of estrogens on ceruloplasmin expression and the sex-specific responses of ceruloplasmin to Cu intake [44,45]. The different association of serum Cu and ceruloplasmin concentrations to the humoral immune response to vaccination points to the potential relevance of non-ceruloplasmin bound Cu, a fraction of circulating Cu that has been associated with oxidative stress, damage, cognitive decline and Alzheimer disease [46]. Alzheimer patients with elevated non-ceruloplasmin bound Cu showed a less severe cerebral atrophy, suggestive of a distinct role of mobile Cu for the disease process [47]. In this respect, the critical role of immune cells for controlling the ratio of free versus protein-bound trace elements, and between their availability to potential pathogens versus host cells merits consideration, as this balance responds dynamically to infections [48]. Notably, ceruloplasmin has been shown to deliver Cu to immune cells, thereby supporting immune responses after infection and immunization, and likely affecting the balance of Cu-free apoenzyme to Cu-loaded ceruloplasmin [49]. As the enzymatic activities of ceruloplasmin depend on the Cu ligand, the balance of Cu-free to Cu-loaded ceruloplasmin determines its ferroxidase activity, iron metabolism, and may be responsible for the herein observed incongruent relationship to the vaccination response in comparison to total serum Cu [50].

### 3.1 Association of copper status and vaccination response

The importance of a replete Cu status for a fully functioning immune system, in particular for the adequate activity of immune cells such as neutrophils, monocytes and T cells, has been demonstrated in several clinical and experimental studies [14,25,51,52]. Serum Cu is known to increase in inflammation and infections, as part of the positive acute phase response [53,54]. A potential permissive effect of Cu on the biosynthesis of antibodies by B cells is less well characterized, and the interaction is mainly known from severe Cu deficiency impairing humoral immune responses in mice [55]. Focused studies analyzing the role of Cu in healthy non-deficient adults have so far been missing, in particular in view of the current pandemic and the importance of efficient vaccination programs [56]. There are only few clinical studies analyzing immune parameters in relation to Cu status. Intervention studies with high and low copper diets have reported positive effects on indices of immune function, with dose-dependent associations [57,58]. These findings are in accordance with our results, indicating that Cu status and immune system activity correlate over wide concentration ranges, and are not only relevant in severe Cu deficiency. Even sub-optimal supply and slight deficiencies may be of relevance for an intensive immune response and an efficient increase of vaccination-induced antibodies.

### 3.2 Combined trace element deficiency and vaccination response

In previous studies, baseline concentrations of the essential trace elements Se and Zn have been associated with the activity of the immune system, collectively indicating the importance of replete supply [6,7,10]. Yet, our prior analyses indicated that the serum Se or Zn concentrations in this group of adult health care workers were unrelated to the antibody response after vaccination [35,36]. This notion accords with the analysis of the group of participants with a combined Se–Zn deficiency (DQ1-Se-Zn) in this study, which was not different from the better supplied subjects. However, once severely diseased and admitted to hospital, a combined deficiency of the trace elements was associated with higher complication rates in pulmonary infections [59]. The diverse observational studies have recently been supported by a randomized intervention trial (RCT), indicating significantly enhanced immune responses in subjects receiving Se and Zn supplementation [60]. This notion has recently been reinforced by the results from an RCT with supplemental Zn, reducing hospital stay and mortality rate in hospitalized patients with severe COVID-19 [61].

Our study accords with these findings on the importance of a sufficiently high trace elements supply, and indicates that Cu, and in particular the combined Cu and Zn status, are of relevance for an efficient immune response both acutely and for longer periods of time post vaccination. As mentioned, our study was not indicating any apparent modifying effect of Se status on these interactions. This finding may be due to the specific cohort studied, as the enrolled healthy adults from the particular clinics in Germany were well-aware of the high importance of a replete Se status for general health, in particular for surviving COVID-19, and almost none of the participants displayed strong Se deficiency at the different time points [36]. This particular pre-requisite is not usually found across Europe, where many subjects display strong Se deficiency [62], in particular when infected and admitted to hospital [29,63].

### 3.3 Strength and limitations

Among the strengths of this study is the longitudinal design with a group of healthy volunteers who were vaccinated according to a pre-specified protocol during a well-defined short period of time. All participants received the same vaccine, and the serum samples were processed by standard operating procedures in parallel. In addition, the laboratory analyses were performed by experienced personnel blinded to all clinical information. Further, the two most informative biomarker of Cu status were determined and correlated linearly, and two meaningful readouts of vaccination-induced antibodies were assessed and compared, yielding congruent results.

Among the notable limitations of the study are the focus on humoral parameters only, and the limited size of the cohort, where the majority of participants were female. Even though, two biomarkers of Cu status were analyzed, information of Cu-free versus Cu-loaded ceruloplasmin was not available, limiting the interpretation on ceruloplasmin enzymatic function under these conditions. In addition, all the participants were vaccinated with the BNT162b2 vaccine, and the results may not be applicable and of relevance to other SARS-CoV-2 vaccines. Moreover, like in all observational studies, residual confounding cannot be ruled out entirely. Information on BMI, smoking, alcohol intake or socioeconomic status, which were shown to possibly associate to Cu status and mortality, were not accessible. Moreover, follow up data on additional vaccinations and potential disease courses upon SARS-CoV-2 infections are unknown and were unfortunately not planned during the design and ethical approval of the study. Finally, the population studied was mainly European females, and extrapolations to other populations with different genetic or geographical background may not be appropriate.

## 4 Conclusion

In conclusion, this study shows that a copper status below 1024.55 μg/L is associated with a reduced vaccination response three weeks after Covid-19 vaccination. Furthermore, participants with a copper status below 1024.55 combined with a zinc status below 764.3 had reduced IgG titres three and six weeks after vaccination.

### 4.1 STAR methods

**Key resources table**.

**Table:** 

Reagent or Resource	Source	Identifier
**Biological samples**		
Healthy donor serum	Klinikum Aschaffenburg-Alzenau	EA No. #20033; ID: DRKS00022294
**Critical commercial assays**		
IDS SARS-CoV-2 IgG	Immunodiagnosticsystems	Cat#CVCL100G
Spike Protein Inhibition Assay (SPIA)	Immunodiagnosticsystems	Cat#DKO205/RUO
**Software and package**		
RStudio integrated development environment	Posit Software	https://posit.co/products/open-source/rstudio/
dplyr	https://cran.r-project.org/web/packages/dplyr/index.html	https://cran.r-project.org/web/packages/dplyr/index.html
tidyr	https://cran.r-project.org/web/packages/tidyr/index.html	https://cran.r-project.org/web/packages/tidyr/index.html
gtsummary	https://cran.r-project.org/web/packages/gtsummary/index.html	https://cran.r-project.org/web/packages/gtsummary/index.html
ggplot2	https://cran.r-project.org/web/packages/ggplot2/index.html	https://cran.r-project.org/web/packages/ggplot2/index.html
ggpubr	https://cran.r-project.org/web/packages/ggpubr/index.html	https://cran.r-project.org/web/packages/ggpubr/index.html
**Other**		
Benzoquinone Activated Horseradish Peroxidase (BQ-HRP)	Immunodiagnosticsystems	Cat#JC-1503-003
BioFx TMB One Component HRP Microwell Substrat	SURMODICS	Cat#TMBW-0100-01
sulphuric acid 96 %	CARL ROTH	Cat#4623.1
Trace Elements Serum L-1	Seronorm	Cat#201405
Gallium standard	fischer scientific	Cat#16674124

### 4.2 Resource availability

#### 4.2.1 Lead contact

Further information and any related requests should be directed to and will be fulfilled by the lead contact, Lutz Schomburg (Lutz.Schomburg@charite.de).

#### 4.2.2 Material availability

This study did not generate new unique reagents.

#### 4.2.3 Study design and subject details

In the prospective observational ATORG study, all blood samples were collected from healthy adult health care workers [35]. The authorities in Bavaria, Germany, provided ethical counselling (Ethik-Kommission der Bayerischen Landesärztekammer, Munich, Germany, EA No. #20033), and the study had been registered at the German Clinical Trial Register (Deutsches Register Klinischer Studien, ID: DRKS00022294, Sept. 14th 2020), with an amendment added Jan. 12th, 2021. All enrolled participants gave written informed consent at the start of the study and consented to the publication of clinical results. The final cohort consisted mainly of female (n = 110) health care workers (n = 126 at baseline), who received two sequential doses of the Biontech/Pfizer vaccine (BNT162b2) in a coordinated vaccination process (Table 1). All enrolled participants were not pregnant and had no pre-existing health conditions. In addition, all Study participants completed a questionnaire about their diet and supplement intake. Serum samples were drawn at four time points, i.e. at the time of first vaccination (n = 126), at the second dose (n = 115) (week three), six weeks after first vaccination (n = 113) and at week 24 (n = 56). All samples obtained were shipped on dry ice to the analytical laboratory in Berlin, Germany, for trace element and immunoglobulin analysis by scientists and technicians blinded to the clinical data.

#### Ethical approval

The study was conducted according to the guidelines of the Declaration of Helsinki, and ethical counselling was provided by the authorities in Bavaria, Germany (Ethik-Kommission der Bayerischen Landesärztekammer, Munich, Germany EA No. #20033). The study was registered at the German Clinical Trial Register (Deutsches Register Klinischer Studien, ID: DRKS00022294, September 14, 2020) with an amendment approved by the Ethik-Kommission der Bayerischen Landesärztekammer, Munich, Germany, on January 12, 2021 (EA No. #20033a).

## 5 Method details

### 5.1 Measurement of antibodies to SARS-CoV-2 and their neutralizing activity

The methods for quantification of SARS-CoV-2 IgG and the assessment of their neutralizing activity were described earlier [34,36]. In brief, the SARS-CoV-2 IgG concentration was determined by a chemiluminescent two-step sandwich immunoassay on the ISYS automated laboratory analyzer (TGS COVID-19 IgG, Cat#CVCL100G, Immunodiagnostic Systems Holdings PLC (ids), UK). According to the manufacturer, the measuring range for the neutralizing activity is 0.0–160.0 AU/mL, with readings above 11.5 AU/mL indicating seropositivity. Samples above the measurable range should be diluted two to sixteen times with the supplied diluent and then corrected by the dilution factor to determine the initial concentration. Neutralization activity against the COVID-19 spike protein was determined via a commercial competitive immune-enzymatic colorimetric method (Cat#DKO205/RUO), as described [34,36]. The measurement range of the kit yields readouts from 0 to 100 %, and according to the manufacturer, interferences of 30 % and above are considered positive.

### 5.2 Trace element quantification

The concentration of serum trace elements was determined via total reflection X-ray fluorescence (TXRF) analysis using a benchtop TXRF spectrometer (S4 T-STAR, Bruker Nano GmbH, Berlin, Germany), as described [64,65]. Briefly, spiked serum samples with a gallium standard (cat#16674124, Merck KGaA, Darmstadt, Germany) (1000 μg/L) (1:2) were applied to polished glass slides and dried at 37 °C. The fluorescence from X-ray activation was captured by the benchtop TXRF spectrometer and used to calculate trace elements concentrations from the emission spectrum. A standard serum with validated trace elements concentrations (cat#201405, Seronorm Sero AS, Billingstad, Norway) served as control; intra- and inter-assay of coefficients of variation were below 5 % during the measurements.

### 5.3 Ceruloplasmin measurement

The concentration of circulating ceruloplasmin was assessed by a two-site non-competitive immunoassay as described earlier [66]. In brief, serum samples were pre-diluted 1:300, and 50 μl aliquots were incubated at room temperature on pre-coated ELISA plates with a ceruloplasmin-specific monoclonal antibody. After 30 min a three-times automatic wash step was performed to rinse the ELISA plates using a HydroFlexTM microplate washer (Tecan Group AG, Maennedorf, Switzerland). For detection, 50 μl of a second ceruloplasmin-specific monoclonal antibody coupled to horseradish peroxidase (cat#JC-1503-003) was added. Enzymatic detection was performed by adding 100 μL of 3,3′,5,5′-tetramethylbenzidine (cat#TMBW-0100-01), and the reaction was stopped by sulphuric acid (cat#4623.1) (0.25 M, 100 μL per well). Spectrophotometric read out was recorded within 10 min at 450 nm using a NanoQuant Infinite 200 Pro microplate reader (Tecan Group AG). Intra- and inter-assay coefficients of variation were below 15 % during the measurements.

### 5.4 Statistical analysis

Baseline characteristics of participants are presented as median (IQR; interquartile range), stratified by sex. Participants were categorized into tertiles (Q1, Q2, Q3) according to baseline status of each trace element or biomarker. Differences in humoral immune response across tertiles were investigated at different time points using Kruskal-Wallis-test. When comparing two groups of independent samples, i.e. in the case of combined deficiencies, Wilcoxon-Rank-sum test was used. Correlation of trace elements with humoral immune response parameters was investigated using Spearman's rank correlation test. All statistical analyses were conducted using the R language, on the RStudio software [67], version 4.1.2, environment. The following packages were implemented into R Studio; dplyr [68], tidyr [69], gtsummary [70], ggplot2 [71], and ggpubr [72].

## Funding

The research has been supported by the 10.13039/501100001659Deutsche Forschungsgemeinschaft (10.13039/501100001659DFG), Research Unit FOR-2558 “TraceAge” (Scho 849/6–2), and 10.13039/501100003383CRC/TR 296 “Local control of TH action” (LocoTact, P17).

## Data availability statement

The data that has been used is confidential. Anonymised data and code for statistical analyses are available upon reasonable request from the corresponding author.

## CRediT authorship contribution statement

**Thilo Samson Chillon:** Conceptualization, Formal analysis, Investigation, Validation, Writing – original draft. **Kamil Demircan:** Data curation, Formal analysis, Methodology, Writing – original draft. **Julian Hackler:** Data curation, Formal analysis, Writing – review & editing. **Raban A. Heller:** Data curation, Formal analysis, Writing – review & editing. **Peyman Kaghazian:** Formal analysis, Resources, Writing – review & editing. **Arash Moghaddam:** Conceptualization, Formal analysis, Resources, Writing – review & editing. **Lutz Schomburg:** Conceptualization, Formal analysis, Funding acquisition, Resources, Supervision, Writing – original draft.

## Declaration of competing interest

The authors declare the following financial interests/personal relationships which may be considered as potential competing interests:L.S. hold shares of selenOmed GmbH, a company involved in Se status assessment.There are no other activities or relationships that may have influenced the submitted work.

## Abbreviations

BNT162b2: BioNTech SARS-CoV-2 vaccine
COX: cytochrome *c* oxidase
COVID-19: coronavirus disease 2019
Cu: Copper
Cu/Zn-SOD: superoxide dismutase
DQ1: Double Q1
ELISA: Enzyme-linked Immunosorbent Assay
Fe: Iron
Ids: Immunodiagnostic Systems Holdings PLC
IgG: Immunoglobulin G
IQR: Median
LOX: lysyl oxidase
mRNA: messenger ribonucleic acid
PAM: peptidylglycine alpha-amidating monooxygenase
RCT: randomized intervention trial
SARS-CoV-2: severe acute respiratory syndrome coronavirus type 2
Se: Selenium
TQ1: Triple Q1
TXRF: total reflection X-ray fluorescence
yr: year
Zn: Zinc

## References

[1] Golin A. (2023). Relationship between selenium status, selenoproteins and COVID-19 and other inflammatory diseases: a critical review. J. Trace Elem. Med. Biol..

[2] Haase H., Schomburg L. (2019). You'd better zinc-trace element homeostasis in infection and inflammation. Nutrients.

[3] Hoffmann P.R., Berry M.J. (2008). The influence of selenium on immune responses. Molecular Nutrition & Food Research.

[4] Munteanu C., Schwartz B. (2022). The relationship between nutrition and the immune system. Front. Nutr..

[5] Shakoor H. (2021). Immune-boosting role of vitamins D, C, E, zinc, selenium and omega-3 fatty acids: could they help against COVID-19?. Maturitas.

[6] Yao Y. (2021). Selenium–GPX4 axis protects follicular helper T cells from ferroptosis. Nat. Immunol..

[7] Girodon F. (1999). Impact of trace elements and vitamin supplementation on immunity and infections in institutionalized elderly patients: a randomized controlled trial. MIN. VIT. AOX. geriatric network. Arch Intern Med.

[8] Rahman M.J. (2005). Effects of zinc supplementation as adjunct therapy on the systemic immune responses in shigellosis. Am. J. Clin. Nutr..

[9] Ahmad S.M. (2016). Maternal zinc supplementation improves hepatitis B antibody responses in infants but decreases plasma zinc level. Eur. J. Nutr..

[10] Kewcharoenwong C. (2022). Daily preventive zinc supplementation increases the antibody response against pathogenic Escherichia coli in children with zinc insufficiency: a randomised controlled trial. Sci. Rep..

[11] Langkamp-Henken B. (2004). Nutritional formula enhanced immune function and reduced days of symptoms of upper respiratory tract infection in seniors. J. Am. Geriatr. Soc..

[12] Linder M.C., Hazegh-Azam M. (1996). Copper biochemistry and molecular biology. Am. J. Clin. Nutr..

[13] Arredondo M., Núñez M.T. (2005). Iron and copper metabolism. Mol. Aspect. Med..

[14] Percival S.S. (1998). Copper and immunity. The American Journal of Clinical Nutrition.

[15] Lockyer S. (2020). Effects of diets, foods and nutrients on immunity: implications for COVID-19?. Nutr. Bull..

[16] Lalioti V. (2009). Molecular mechanisms of copper homeostasis. FBL.

[17] Camakaris J., Voskoboinik I., Mercer J.F. (1999). Molecular Mechanisms of Copper Homeostasis. Biochemical and Biophysical Research Communications.

[18] Ishida S. (2013). Bioavailable copper modulates oxidative phosphorylation and growth of tumors. Proc Natl Acad Sci U S A.

[19] Yin P. (2011). Probing the production of amidated peptides following genetic and dietary copper manipulations. PLoS One.

[20] D'Alessandro A., Zolla L. (2011). The SODyssey: superoxide dismutases from biochemistry, through proteomics, to oxidative stress, aging and nutraceuticals. Expert Rev. Proteomics.

[21] Linder M.C. (2021). Apoceruloplasmin: abundance, detection, formation, and metabolism. Biomedicines.

[22] Helman S.L. (2023). The biology of mammalian multi-copper ferroxidases. Biometals.

[23] Pocino M., Baute L., Malavé I. (1991). Influence of the oral administration of excess copper on the immune response. Fund. Appl. Toxicol..

[24] Massie H.R., Ofosu-Appiah W., Aiello V.R. (1993). Elevated serum copper is associated with reduced immune response in aging mice. Gerontology.

[25] Bonham M. (2002). The immune system as a physiological indicator of marginal copper status?. Br. J. Nutr..

[26] Failla M.L., Hopkins R.G. (1998). Is low copper status immunosuppressive?. Nutr. Rev..

[27] Liu Y. (2022). Copper regulation of immune response and potential implications for treating orthopedic disorders. Front. Mol. Biosci..

[28] Hackler J. (2021). Relation of serum copper status to survival in COVID-19. Nutrients.

[29] Demircan K. (2022). Association of COVID-19 mortality with serum selenium, zinc and copper: six observational studies across Europe. Front. Immunol..

[30] Govind V. (2021). Antiviral properties of copper and its alloys to inactivate covid-19 virus: a review. Biometals.

[31] Deloncle R. (2022). Copper acetate aerosols: a possible tool complementary to vaccination in fight against SARS-CoV-2 and variants replication. Med. Hypotheses.

[32] Takeda Y. (2021). Application of copper iodide nanoparticle-doped film and fabric to inactivate SARS-CoV-2 via the virucidal activity of cuprous ions (Cu(+)). Appl. Environ. Microbiol..

[33] Tateyama-Makino R. (2021). The inhibitory effects of toothpaste and mouthwash ingredients on the interaction between the SARS-CoV-2 spike protein and ACE2, and the protease activity of TMPRSS2 in vitro. PLoS One.

[34] Chillon T.S. (2021). Relationship between vitamin D status and antibody response to COVID-19 mRNA vaccination in healthy adults. Biomedicines.

[35] Chillon T.S. (2022). Serum free zinc is associated with vaccination response to SARS-CoV-2. Front. Immunol..

[36] Demircan K. (2022). Humoral immune response to COVID-19 mRNA vaccination in relation to selenium status. Redox Biol..

[37] Lustig Y. (2021). BNT162b2 COVID-19 vaccine and correlates of humoral immune responses and dynamics: a prospective, single-centre, longitudinal cohort study in health-care workers. The Lancet Respiratory Medicine.

[38] Turley C.B. (2023). Modifiers of COVID-19 vaccine efficacy: results from four COVID-19 prevention network efficacy trials. Vaccine.

[39] Baudry J. (2020). Changes of trace element status during aging: results of the EPIC-Potsdam cohort study. Eur. J. Nutr..

[40] Cabral M. (2021). Trace element profile and incidence of type 2 diabetes, cardiovascular disease and colorectal cancer: results from the EPIC-Potsdam cohort study. Eur. J. Nutr..

[41] Ghayour-Mobarhan M. (2005). Determinants of serum copper, zinc and selenium in healthy subjects. Ann. Clin. Biochem..

[42] Johnson P.E., Milne D.B., Lykken G.I. (1992). Effects of age and sex on copper absorption, biological half-life, and status in humans. Am. J. Clin. Nutr..

[43] Grandjean P. (1992). Reference intervals for trace elements in blood: significance of risk factors. Scandinavian Journal of Clinical and Laboratory Investigation.

[44] Méndez M.A. (2004). Sex and ceruloplasmin modulate the response to copper exposure in healthy individuals. Environ. Health Perspect..

[45] Clemente C. (1992). Ceruloplasmin serum level in post-menopausal women treated with oral estrogens administered at different times. Horm. Metab. Res..

[46] Squitti R. (2019). Copper in glucose intolerance, cognitive decline, and alzheimer disease. Alzheimer Dis. Assoc. Disord..

[47] Squitti R. (2017). Patients with increased non-ceruloplasmin copper appear a distinct sub-group of alzheimer's disease: a neuroimaging study. Curr. Alzheimer Res..

[48] Healy C. (2021). Nutritional immunity: the impact of metals on lung immune cells and the airway microbiome during chronic respiratory disease. Respir. Res..

[49] Collins J.F. (2021). Copper nutrition and biochemistry and human (patho)physiology. Adv. Food Nutr. Res..

[50] Helman S.L. (2023). The biology of mammalian multi-copper ferroxidases. Biometals.

[51] Wintergerst E.S., Maggini S., Hornig D.H. (2007). Contribution of selected vitamins and trace elements to immune function. Ann. Nutr. Metabol..

[52] Minatel L., Carfagnini J.C. (2000). Copper deficiency and immune response in ruminants. Nutr. Res..

[53] Hackler J. (2020). Copper and selenium status as biomarkers of neonatal infections. J. Trace Elem. Med. Biol..

[54] Wang B., Wang X.P. (2019). Does ceruloplasmin defend against neurodegenerative diseases?. Curr. Neuropharmacol..

[55] Prohaska J.R., Lukasewycz O.A. (1981). Copper deficiency suppresses the immune response of mice. Science.

[56] Galmés S., Serra F., Palou A. (2020). Current state of evidence: influence of nutritional and nutrigenetic factors on immunity in the COVID-19 pandemic framework. Nutrients.

[57] Kelley D.S. (1995). Effects of low-copper diets on human immune response. Am. J. Clin. Nutr..

[58] Turnlund J.R. (2004). Long-term high copper intake: effects on indexes of copper status, antioxidant status, and immune function in young men. Am. J. Clin. Nutr..

[59] Berger M.M. (2006). Reduction of nosocomial pneumonia after major burns by trace element supplementation: aggregation of two randomised trials. Crit. Care.

[60] Rodriguez J.A.M. (2021). Effect and tolerability of a nutritional supplement based on a synergistic combination of β-glucans and selenium- and zinc-enriched Saccharomyces cerevisiae (ABB C1®) in volunteers receiving the influenza or the COVID-19 vaccine: a randomized, double-blind, placebo-controlled study. Nutrients.

[61] Ben Abdallah S. (2023). Twice-daily oral zinc in the treatment of patients with coronavirus disease 2019: a randomized double-blind controlled trial. Clin. Infect. Dis..

[62] Stoffaneller R., Morse N.L. (2015). A review of dietary selenium intake and selenium status in Europe and the Middle East. Nutrients.

[63] Foshati S. (2022). Antioxidants and clinical outcomes of patients with coronavirus disease 2019: a systematic review of observational and interventional studies. Food Sci. Nutr..

[64] Hoeflich J. (2010). The choice of biomarkers determines the selenium status in young German vegans and vegetarians. Br. J. Nutr..

[65] Jeffery J. (2019). Method for measurement of serum copper, zinc and selenium using total reflection X-ray fluorescence spectroscopy on the PICOFOX analyser: Validation and comparison with atomic absorption spectroscopy and inductively coupled plasma mass spectrometry. Ann. Clin. Biochem..

[66] Hackler J. (2020). Copper and selenium status as biomarkers of neonatal infections. J. Trace Elem. Med. Biol..

[67] Ross I., Robert G. (1996). Radiokhimiya: A Language for Data Analysis and Graphics. Journal of Computational and Graphical Statistics.

[68] Wickham H. (2015). dplyr: A grammar of data manipulation. R package.

[69] Wickham H., Henry L. (2016). Easily tidy data with’spread ’and “gather” functions. CRAN R Package.

[70] Sjoberg D.D. (2020). gtsummary: presentation-ready data summary and analytic result tables. R package version.

[71] Wickham H. (2011). ggplot2. WIREs Computational Statistics.

[72] Kassambara A. (2020). ggpubr:“ggplot2” based publication ready plots. R package version 0.4.

