# Supplementary material for: Combined copper and zinc deficiency is associated with reduced SARS-CoV-2 immunization response to BNT162b2 vaccination
Source: Heliyon. 2023 Oct 14;9(10):e20919. doi: 10.1016/j.heliyon.2023.e20919 (PMC10597833; doi:10.1016/j.heliyon.2023.e20919)
Supplement: Multimedia component 1 [file mmc1.docx]

**Supplementary File**

**Combined copper and zinc deficiency is associated with reduced SARS-CoV-2 immunization response to BNT162b2 vaccination**

**Authors:** Thilo Samson Chillon^1^, Kamil Demircan^1^, Julian Hackler^1^, Raban A. Heller^1,2^, Peyman Kaghazian^3^ , Arash Moghaddam^3^, Lutz Schomburg^1^*

**Affiliations:**

^1^ Max Rubner Center for Cardiovascular Metabolic Renal Research (CMR), Institute for Experimental Endocrinology, Charité-Universitätsmedizin Berlin, Hessische Straße 3-4, D-10115 Berlin, Germany.

^2^ Bundeswehr Hospital Berlin, Clinic of Traumatology and Orthopaedics, D-10115 Berlin, Germany.

^3^ Orthopedic and Trauma Surgery, Frohsinnstraße 12, D-63739 Aschaffenburg, Germany

^*^**Contact information for corresponding author**: Max Rubner Center for Cardiovascular Metabolic Renal Research (CMR), Institut für Experimentelle Endokrinologie, Charité-Universitätsmedizin Berlin, Hessische Straße 3-4, D-10115 Berlin, Germany.

Tel: +49 30 450 524 289

Fax: +49 30 450 7524 289

Email address: [Lutz.Schomburg@charite.de](mailto:Lutz.Schomburg@charite.de) (L. Schomburg)

**
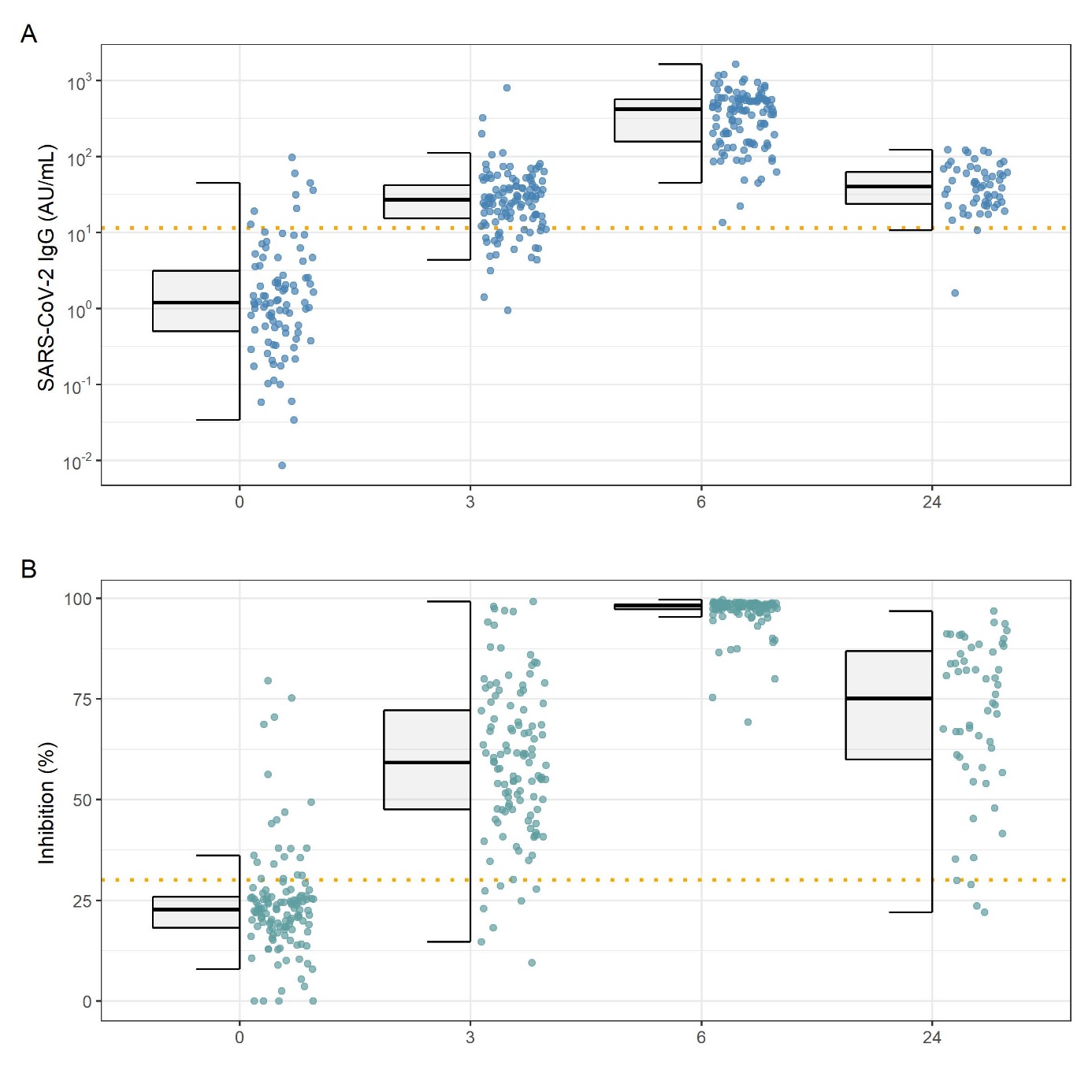
Supplementary Figure 1. SARS-CoV-2 IgG and neutralizing antibody response over 24 weeks in health care workers vaccinated with BNT162b2 vaccine.** **A** **At the time of enrolment, almost all participants were seronegative with titres below the cut-off of 11.5 AU/mL, and 35 patients had no measurable IgG at the start of the study. IgG titres were highest at the 3 week time point after the second dose (week 6) and showed a declining trend towards the 24 week time point. B Almost all of the participants had no detectable neutralizing antibodies at the start of treatment, with titres below the cut-off of 30% inhibition. The inhibition of binding of the spike protein to Angiotensin-converting enzyme 2 was highest at 3 weeks after the second dose (week 6) and showed a decreasing trend towards the 24-week time point.**


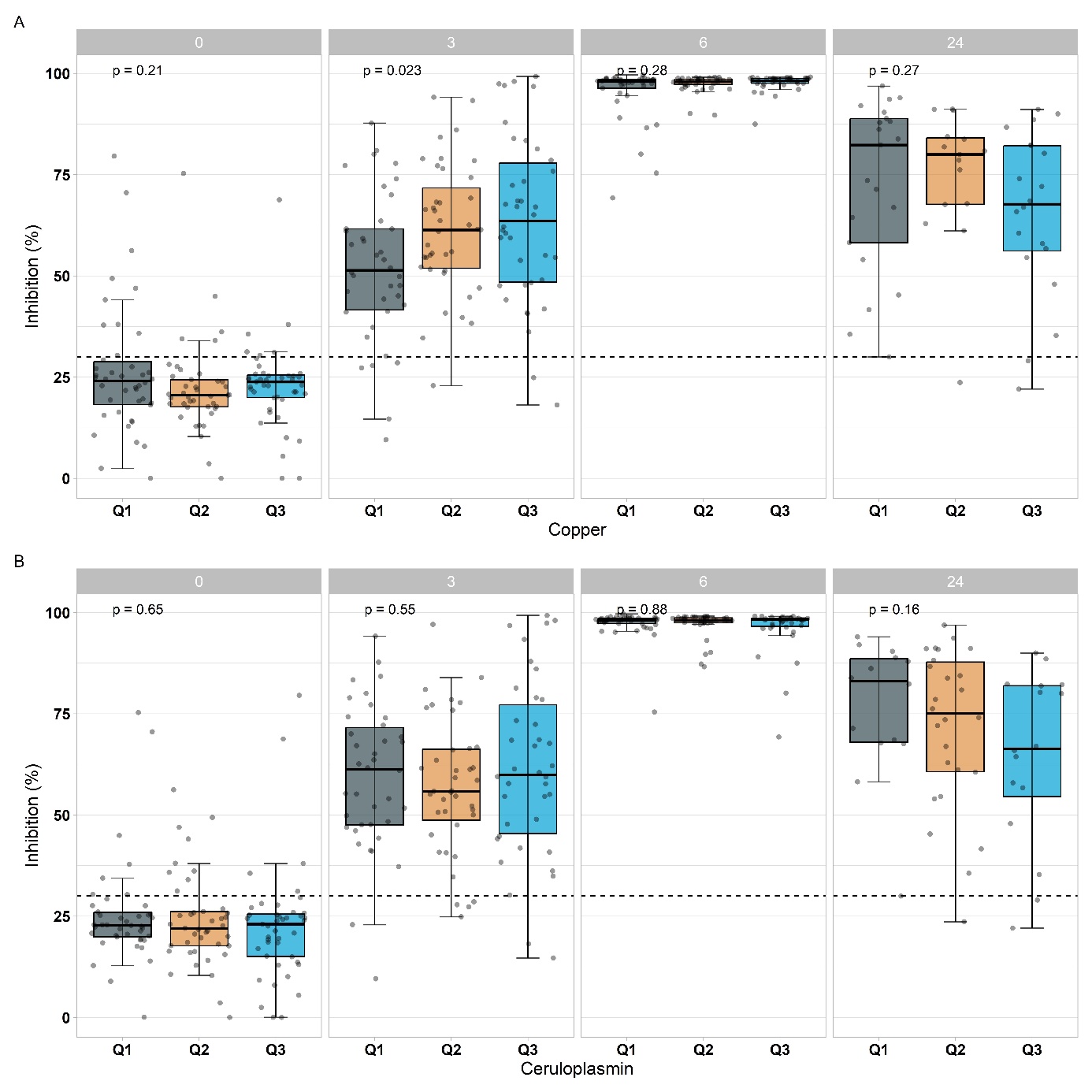


**Supplementary Figure 2. Neutralization activity of serum against SARS-CoV-2 in relation to baseline copper and ceruloplasmin status. A The comparison of neutralizing activity in relation to the different baseline copper status (Q1<1024.55 µg/L; Q2 1024.55 -1211 µg/L; Q3 >1211 µg/L)** at baseline and **in week 3, 6, and 24 does not indicate significant differences. B The neutralizing activity of the serum samples from subjects with different baseline ceruloplasmin status (Q1<340.70 mg/L; Q2 340.70 -453.23 mg/L; Q3 >453.23 mg/L) in week 3, 6, and 24 was not different. Two-sided Kruskal-Wallis test was used to assess differences.**


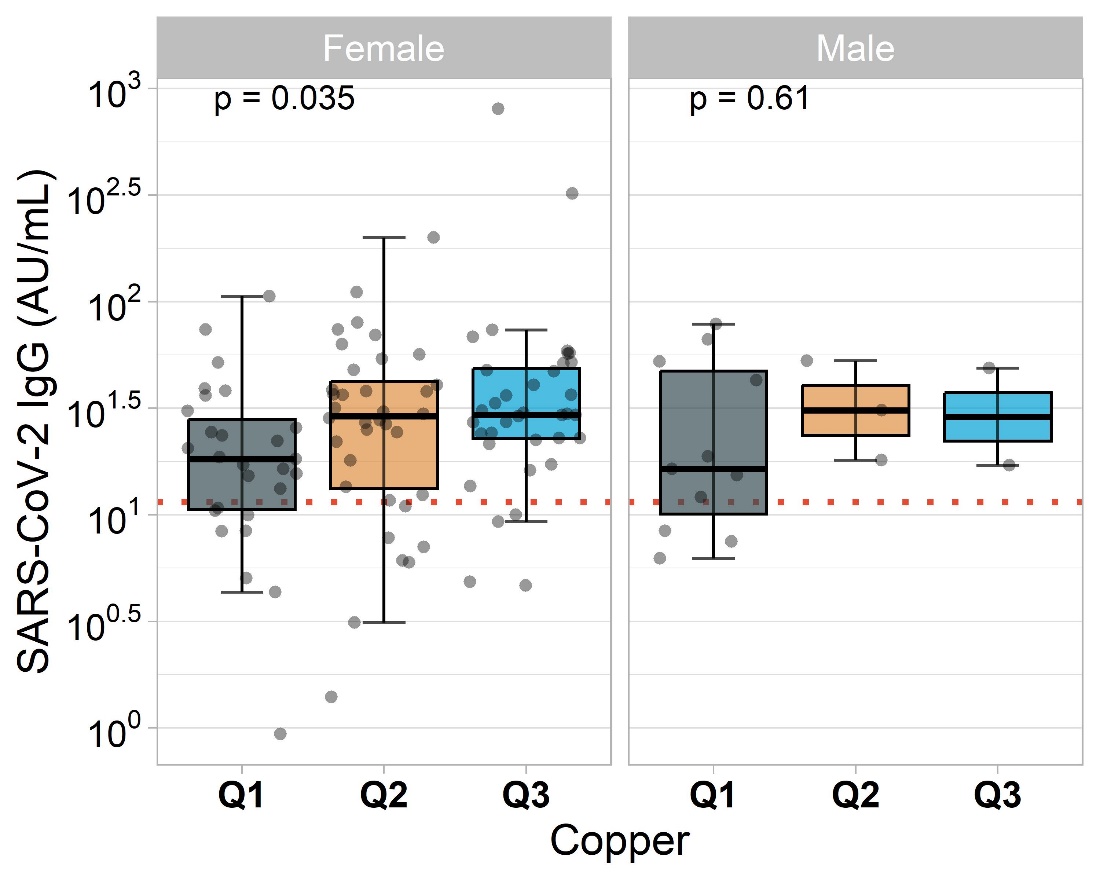


**Supplementary Figure 3. Dynamic changes in SARS-CoV-2 IgG concentrations according to baseline copper status and gender at time point 3 weeks after first vaccination. The comparison of neutralizing activity in relation to the different baseline copper status and gender (Q1<1024.55 µg/L; Q2 1024.55 -1211 µg/L; Q3 >1211 µg/L) at week 3 after first vaccination, indicates significant differences in females only.**


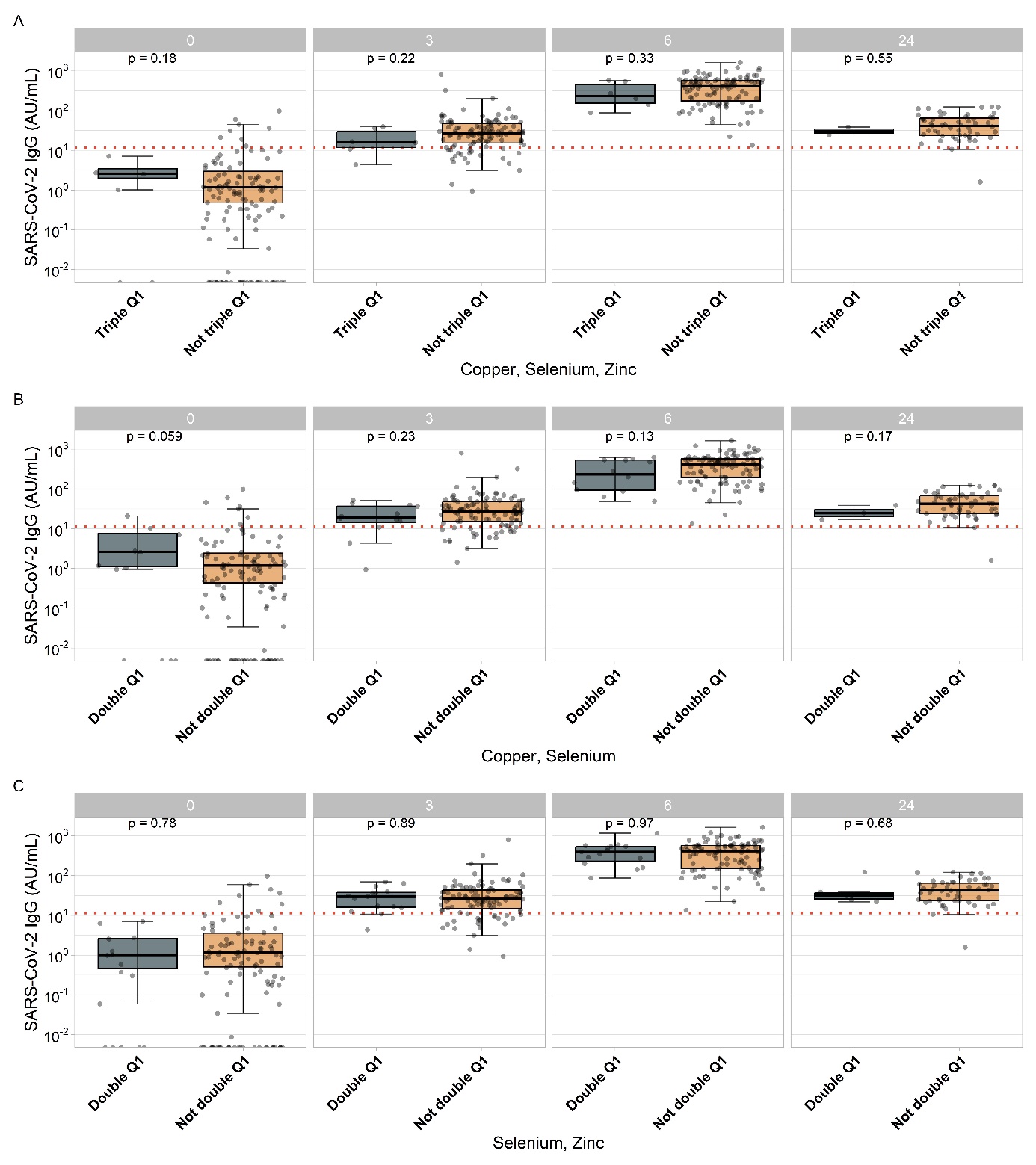


**Supplementary Figure 4. SARS-CoV-2 IgG according to triple Q1 and double Q1 at baseline. A** Comparison of SARS-CoV-2 IgG of participants with triple Q1, in the trace elements copper, selenium and zinc at baseline, to not triple Q1 deficiency at baseline and in week 3, 6, and 24, shows no differences (Triple Q1: Cu <1024.55; Se <70.8; Zn < 764.3 µg/L). **B** Comparison of SARS-CoV-2 IgG of participants with double Q1, in the TE copper and selenium at baseline, to not double Q1 deficiency in week 3, 6, and 24, shows no differences (Double Q1: Cu <1024.55; Se <70.8 µg/L). **C** Comparison of SARS-CoV-2 IgG of participants with double Q1, in the trace elements selenium and zinc at baseline, to not double Q1 deficiency in week 3, 6, and 24, shows no differences (Double Q1: Zn < 764.3; Se <70.8 µg/L). Pairwise comparisons were conducted by applying the Wilcoxon-Rank-Sum test.


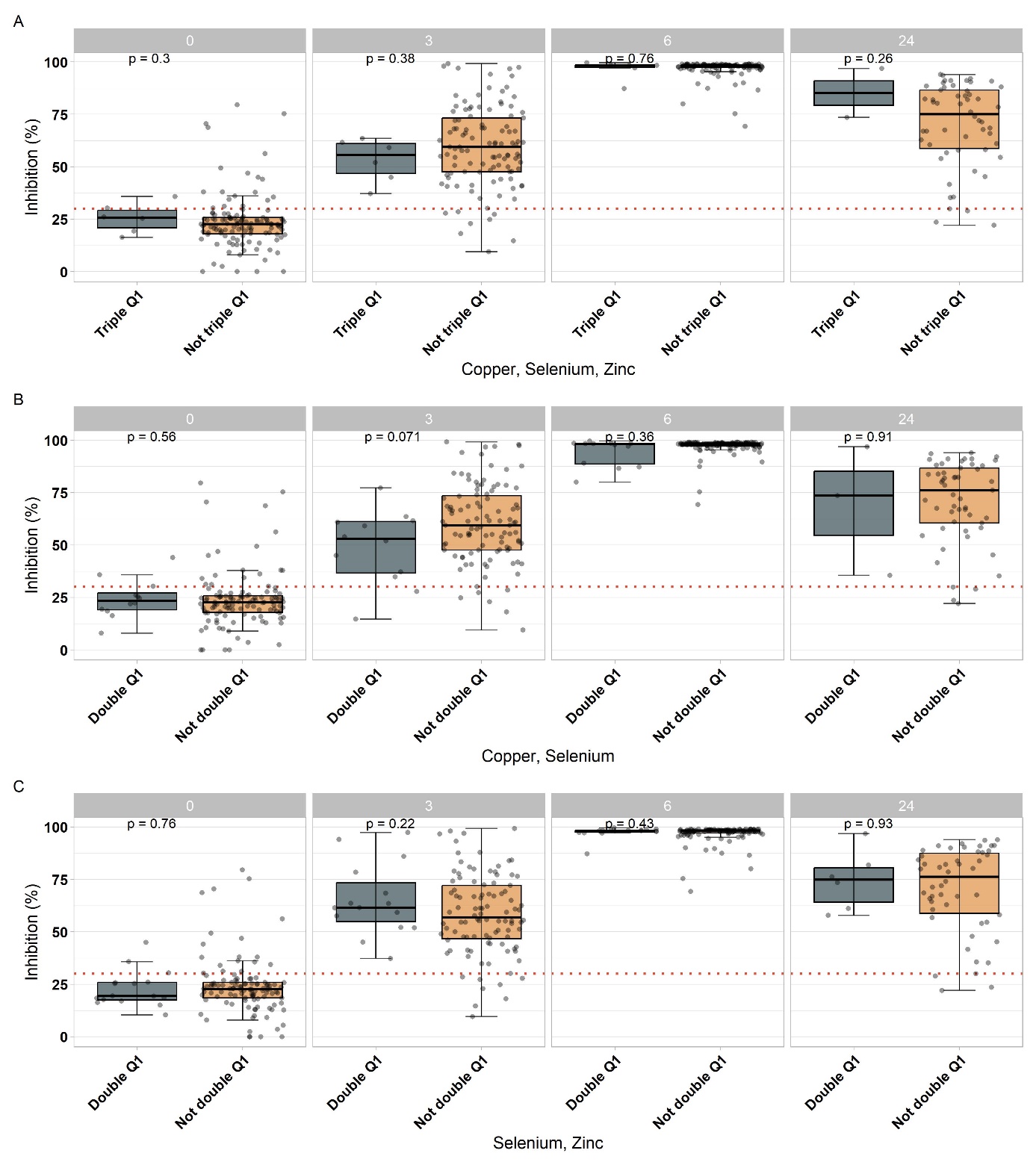


**Supplementary Figure 5. SARS-CoV-2 neutralization activity according to triple Q1 and double Q1 deficiency at baseline. A** Comparison of neutralizing activity of participants with triple Q1 deficiency, in the trace elements copper, selenium and zinc simultaneously at baseline, to not triple Q1 deficiency in week 3, 6, and 24, shows no significant differences. (Triple Q1: Cu <1024.55; Se <70.8; Zn < 764.3 µg/L). B Comparison of neutralizing activity of participants with double Q1 deficiency, in the trace elements copper and selenium simultaneously at baseline, to not double Q1 deficiency in week 3, 6, and 24, shows no significant differences. (Double Q1: Cu <1024.55; Se <70.8 µg/L). C Comparison of neutralizing activity of participants with double Q1 deficiency, in the trace elements selenium and zinc simultaneously at baseline, to not double Q1 deficiency in week 3, 6, and 24, shows no significant differences (Double Q1: Zn < 764.3; Se <70.8 µg/L). Pairwise comparisons were conducted by applying the Wilcoxon-Rank-Sum test.
